# *In vitro* Studies and Clinical Observations Imply a Synergistic Effect Between Epstein-Barr Virus and Dengue Virus Infection

**DOI:** 10.3389/fmicb.2021.691008

**Published:** 2021-06-18

**Authors:** Xiao-Mei Deng, Ling-Zhai Zhao, Xue-Ying Liang, Dan Li, Lei Yu, Fu-Chun Zhang, Hua Zhang, Zhong-Yu Liu, Pei Xu

**Affiliations:** ^1^The Centre for Infection and Immunity Studies, School of Medicine, Sun Yat-sen University, Guangzhou, China; ^2^Guangzhou Eighth People’s Hospital, Guangzhou Medical University, Guangzhou, China

**Keywords:** dengue virus, dengue fever, Epstein–Barr virus, coinfection, reactivation

## Abstract

Dengue virus (DENV) infection can lead to a complex spectrum of clinical outcomes, ranging from asymptomatic infection to life-threatening severe dengue. The reasons for thus drastically varying manifestations of the disease remain an enigma. Herein, we reported an original discovery of the synergistic effect between preexisting Epstein–Barr virus (EBV) infection and DENV superinfection *in vitro* and of a strong correlation of these two viruses in the clinical samples from dengue patients. We showed that (I) DENV-2 infection of an EBV-positive cell line (EBV + Akata cell) reactivated EBV, and it could be blocked by wortmannin treatment. (II) Examination of human peripheral blood mononuclear cell (PBMC) samples from dengue patients revealed significantly elevated cell-associated EBV DNA copy number at the time of hospitalization vs. at the time of disease recovery in most individuals. (III) EBV infection promoted DENV propagation in both EBV-hosting B cells and indirectly in THP-1 cells, supported by the following evidence: (A) EBV + Akata cells were more permissive to DENV-2 infection compared with Akata cells harboring no EBV virus (EBV- Akata cells). (B) Low-molecular weight fraction secreted from EBV + Akata cells could enhance DENV-2 propagation in monocytic THP-1 cells. (C) While reactivation of EBV in EBV + Akata cells further increased DENV-2 yield from this cell line, pharmacological inhibition of EBV replication by acyclovir had the opposite effect. To our knowledge, this is the first investigation demonstrating a positive correlation between EBV and DENV *in vitro* and in human biospecimens.

## Introduction

Dengue virus (DENV) serotypes 1–4 (DENV-1–DENV-4) caused approximately 390 million human infections globally per year; about 96 million are symptomatic (Bhatt et al., 2013). With no specific antiviral therapies or efficient vaccines available, DENV poses a great threat to human health. Starting through a mosquito bite, Langerhans cells in the epidermis (Wu et al., 2000) and dendritic cells, monocytes, and macrophages in the dermis (Schmid and Harris, 2014) are likely the primary targets of DENV. While circulating monocytes are believed to play a critical role in *in vivo* spread and pathogenesis of DENV (Jessie et al., 2004; Durbin et al., 2008), primary B and T lymphocytes are also shown to be permissive to DENV infection (Lin et al., 2002; Silveira et al., 2018). DENV infection can be asymptomatic or lead to diverse clinical manifestations, ranging from self-limiting dengue fever to life-threatening severe dengue (WHO, 2009). Though previous studies have shown that both host and virus factors, especially the host immunity status, can influence the outcomes of DENV infection (Katzelnick et al., 2017; Robinson and Einav, 2020), investigations in whether and how persistent infections in human hosts contribute to DENV infection were rare.

Epstein–Barr virus (EBV), of the γ-herpesvirus subfamily, is a highly prevalent pathogen (Cohen, 2000; Crawford, 2001). More than 90% of the global population is estimated to be infected by the age of 35 and carries the virus for the rest of life, mostly being latently infected (Ohga et al., 2002; Torniainen-Holm et al., 2018). The life cycle of the virus includes a lytic replication phase, predominantly occurring in oropharyngeal epithelial cells, and a latent infection phase, during which the viral genomes persist in naïve, and memory B cells through attaching to the host chromosomes as episomes (Hochberg et al., 2004). Though the majority of viral genomes are transcriptionally quiescent during latency, a few EBV viral promoters are under dynamic regulation at different stages of latency, expressing distinct sets of viral genes in a cell-dependent manner (Woisetschlaeger et al., 1990; Hughes et al., 2011). Intermittently, latent EBV genomes spontaneously reactivate or are reactivated by various physiological stimuli, including other pathogenic infections. Previously, the interplays between EBV and a number of human pathogens have been reported to contribute to disease outcomes. For instance, it is long known that HIV infection caused aberrantly higher EBV loads in peripheral blood of infected individuals, ultimately leading to frequent development of EBV-associated diseases (Dolcetti et al., 1995; Van Baarle et al., 2002; Vaghefi et al., 2006; Stevens et al., 2007; Petrara et al., 2012; Hernandez et al., 2018; Yan et al., 2018). Investigations in South Africa also revealed a direct molecular link between malaria infection and EBV reactivation in circulating mononuclear cells in affected children (Chene et al., 2007; Njie et al., 2009; Reynaldi et al., 2016), and coinfection of EBV and HCV had been reported to dampen immune responses in hospitalized patients (Shoman et al., 2014).

The convergence of EBV and DENV on their tropism of the immune cells inspired the subsequent investigation. In this study, we investigated the interplay between EBV and DENV using *in vitro* systems and clinical samples, and the findings are (I) DENV-2 replication in EBV-positive B cell lines reactivated EBV and/or promoted EBV replication; (II) PBMC-associated EBV copy numbers were significantly elevated at the symptomatic period of dengue patients; (III) preexisting EBV infection could directly facilitate DENV-2 replication in B cells and indirectly promote DENV-2 growth in a monocyte cell line; and (IV) inhibition of EBV replication by acyclovir (ACV) decreased DENV-2 replication in EBV + Akata cells. Taken together, our results highlighted a synergistic effect between EBV and DENV: the two evolutionarily unrelated human viruses and the association between the EBV–DENV interaction and dengue disease outcomes demand further investigations.

## Materials and Methods

### Cell Lines and Viruses

Burkitt lymphoma (BL)-derived EBV-positive Akata (EBV + Akata), EBV-negative Akata (EBV- Akata), THP-1, BHK-21 cells, Namalwa, and mosquito C6/36 cells were grown as instructed by the American Type Culture Collection (ATCC). The EBV + Akata cell line was persistently infected by a recombinant EBV strain, which carries the green fluorescent protein (GFP) reporter gene and neomycin resistance gene as previously reported (Molesworth et al., 2000; Guerreiro-Cacais et al., 2007).

The DENV-2 (strain NGC) and Zika virus (ZIKV and strain MR766) were prepared using C6/36 cells (Wen et al., 2018). Recombinant vesicular stomatitis virus carrying GFP gene (VSV-GFP) (Fernandez et al., 2002) was amplified in Vero cells. DENV/ZIKV was titrated in BHK-21, and VSV-GFP was titrated in Vero cells by plaque assay as described previously (Lin et al., 2002) and stored at −80°C as single-used aliquots. All experiments involving infectious materials were performed under BSL-2 conditions, following the standard biosafety regulations of the School of Medicine, Sun Yat-sen University.

### Human Biospecimens

Blood samples were collected from hospitalized dengue patients or healthy donors, and peripheral blood mononuclear cells (PBMCs) were isolated by density gradient centrifugation. The samples of the 11 dengue patients were collected in the year 2014 or 2017 when they were hospitalized at the Guangzhou Eighth People’s Hospital. All patients and donors had signed the informed consent form before the samples were collected. This study was ethically approved by the institutional review broad of the School of Medicine, Sun Yat-sen University (No. 2020-0012) and the ethics review board of the Guangzhou Eighth People’s Hospital (No. 2013-1224 and 2016-0264). More information was listed in Supplementary Table 2.

### Virus Infection

Cells were inoculated with viruses at a desired multiplicity of infection (MOI) at 37°C for 2 h. Virus inoculum was subsequently removed, and cells were washed with sterile phosphate-buffered saline (PBS) three times.

### Quantitative Real-Time PCR

DNA from PBMCs was extracted using the DNeasy Blood & Tissue Kit (Qiagen, Hilden, Germany). Total DNA and RNA from cell lines was extracted using E.Z.N.A. Tissue DNA Kit I or E.Z.N.A. Total RNA Kit I (Omega, Bienne, Switzerland).

Epstein–Barr virus genome load in human PBMC samples was determined using Taqman probes targeting *Bam*HI W fragment of EBV genome. Absolute EBV genome copy number was calculated by referring to standards running parallel. Standards: A pcDNA3.1-derived plasmid containing the *Bam*HI W fragment of EBV or EBV-DNA isolated from the Namalwa cells was 10 or 2-fold serial diluted and running in triplicate. The Ct values of glyceraldehyde 3-phosphate dehydrogenase (GAPDH) DNA copy number in the isolated PBMC DNA samples were determined by qPCR for normalization of random variations. EBV genome copy numbers were presented in figures as absolute copy number per 1 × 10^6^ PBMCs.

In brief, total DNA was extracted from 1 × 10^6^ PBMCs and eluted into 200 μl of elution buffer. Then, 8.3 μl of DNA was used for qPCR in triplicate. To calculate the absolute copy number of EBV genome per 1 × 10^6^ PBMCs in patient samples, the following equation was used:

EBVabsolutecopynumber/1×10P6BMCcells=10∧(-0.2743×Ct+10.17)×2008.3×2-ΔCt

Relative EBV genome copy number was quantified using probes targeting EBNA1 by qPCR with TB Green Premix Ex Taq II (Takara). The abundance of EBNA1 was normalized to GAPDH. For gene expression analysis, 2 μg of total RNA was reverse-transcribed into cDNA using Evo M-MLV RT for PCR Kit (Accurate Biology). The expression level of target viral genes was normalized to 18S rRNA. qPCR was performed using StepOnePlus Applied Biosystems. Primers and probe information for qPCR are listed in Supplementary Table 1.

Dengue virus loads in patient samples were determined using the OneStep Real-Time RT-PCR Kit for DENV (Da’an Gene) following the manufacturer’s instructions.

### THP-1 Simulation and Infection Experiment

Here, 1 × 10^5^ cells/ml of EBV + Akata or EBV- Akata cells were cultured for 2 days. The culture medium was collected and cleared by centrifugation at 300 ×*g* × 10 min, followed by 3,000 × *g* × 10 min, and further separated into <100 kDa and >100 kDa fractions by ultrafiltration using Amicon Ultra-15 (Merck, Millipore). Ultrafiltration was performed by 4,000 × *g* × 3 min at 4°C. Then, 2 × 10^6^/ml THP-1 cells were incubated with 0.5 ml of freshly prepared retained fraction and passing-through fraction for 8 h prior to DENV-2 infection or culture medium for 2 h prior to ZIKV infection.

### Neutralizing of DENV-2 Infectivity by Monoclonal Antibody

Here, 0.6 ml of DENV-2 stock (2.5 × 10^6^ PFU/ml) was incubated with 20 μl of anti-DENV E protein neutralizing monoclonal antibody (mAb) NIH1 + NL1 (1.75 mg/ml; Guangzhou Eighth People’s Hospital) at 37°C for 1 h. The efficacy of neutralization was evaluated by plaque assay.

### Western Blot

Western blot was conducted as previously described (Xu et al., 2016). Anti-flavivirus E protein mAb 4G2 (1:6,000 diluted, Merck) and anti-β-actin antibody (1:1,000 diluted, ZSGB-BIO) were used as the primary antibody to detect the corresponding antigen.

### Acyclovir Treatment of EBV + Akata Cells

Here, 2 × 10^6^ cells/ml of EBV + Akata cells were firstly treated with RPMI only or with 0.8% goat anti-human immunoglobulin G serum (Shuangliu Zhenglong Biochem Lab) in RPMI 1640 for 6 h to stimulate EBV reactivation. Then, the medium was replaced with normal culture medium, and aliquots of cells were treated with dimethyl sulfoxide (DMSO) only or ACV MedChemExpress (MCE) at 100 μM for 72 h. EBV genome copy number in different experimental groups in Figure 5A was quantified by qPCR at this point. In Figure 5C, the cells were further centrifuged, washed three times with PBS to remove ACV, and then infected with DENV-2. In Figure 5B, to test the effect of ACV on DENV-2 replication, EBV- Akata cells were pretreated with DMSO or ACV for 72 h before infection with DENV-2 at a MOI of 10. The DENV titer in culture medium at 24 h post-infection (hpi) and 36 hpi was determined by plaque assay.

### Fluorescence Microscopy and Flow Cytometry

Cells were photographed using Olympus CKX53 fluorescence microscope at 72 h post-DENV-2 infection and then collected by centrifuging at 300 × *g* for 10 min. The collected cells were fixed with 4% paraformaldehyde (PFA) before being analyzed by flow cytometer CytoFLEX. GFP was a reporter gene carried by EBV virus in Akata EBV + cells.

### Statistical Analysis

Student’s *t*-test (paired, two-tailed) was used to analyze the statistical significance of changes of EBV copy number in human PBMCs between onset samples and recovery samples in Figure 3D, and Student’s *t*-test (unpaired, two-tailed) was used for other statistical analyses.

## Results

### DENV-2 Infection of EBV + Akata Cells Leads to an Increment of Cell-Associated EBV Genome Copy Number and EBV Reactivation

**FIGURE 1:**
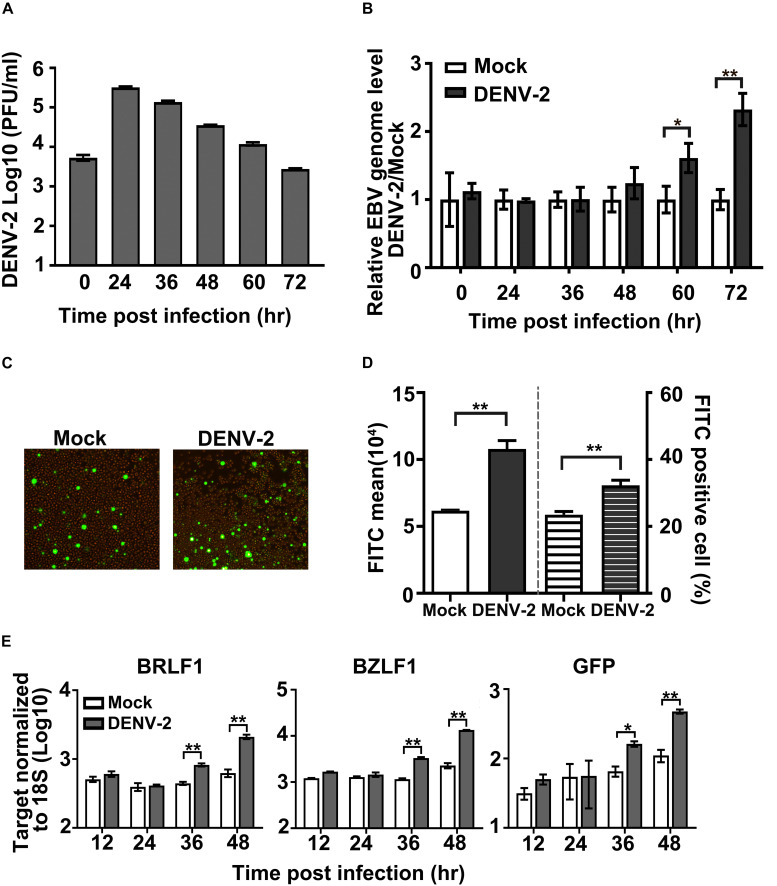
Effect of DENV-2 infection on Epstein–Barr virus (EBV) reactivation in EBV + Akata cells. **(A–E)** EBV + Akata cells were mock-infected or infected with DENV-2 at a multiplicity of infection (MOI) of 10. At the indicated hour post-infection (hpi), DENV-2 titers in the culture medium were determined by plaque assay **(A)**. Cell-associated EBV genome copy number was measured by qPCR and normalized to glyceraldehyde 3-phosphate dehydrogenase (GAPDH). DENV-2-infected groups were normalized to mock-infected group (set as 1) at all time points post-infection and plotted as in panel **B**. At 72 hpi, green fluorescent protein (GFP) level in mock or DENV-2-infected EBV + Akata cells was photographed under a fluorescence microscope **(C)** or quantified by flow cytometer **(D)**. The empty and filled columns on the left half of the chart in panel **D** represented fluorescein isothiocyanate (FITC) mean value, and the striped columns on the right half represented the percentage of FITC-positive cells. mRNA level of BRLF1, BZLF1, and GFP at the indicated time points post-dengue infection was quantified by RT-qPCR, normalized to 18S rRNA, and plotted **(E)**. *p* < 0.01 was marked as ** and *p* < 0.05 was marked as *.

To study the interplay between EBV and DENV, an EBV-positive Burkitt’s lymphoma-derived cell line (EBV + Akata cell) and its EBV-free counterpart (EBV- Akata cell) were utilized as an *in vitro* model system (Shimizu et al., 1994). Firstly, EBV + Akata cells were infected with DENV-2 at a MOI of 10, the culture medium was collected at different hpi, and DENV-2 yields were titrated by plaque assay. At 24 hpi, the DENV-2 titer increased by approximately 100-fold compared with 0 hpi, then gradually decreased over the next 48 h. The above results suggested an active propagation of DENV-2 in EBV + Akata cells (Figure 1A).

Next, the influence of DENV-2 replication on the preexisting EBV infection was characterized. EBV + Akata cells infected with DENV-2 were collected at the indicated time points, and cell-associated EBV genome copy number was quantified by qPCR. From 60 to 72 hpi, the EBV genome copy number in the DENV-2-infected EBV + Akata cells was significantly higher than that of the uninfected controls (set as 1) (Figure 1B).

Green fluorescent protein expression level in DENV-2-infected EBV + Akata cells was then examined by fluorescence microscopy and flow cytometry, since it served as an indicator for EBV reactivation and active replication in this cell line (Guerreiro-Cacais et al., 2007). In Akata EBV + cells, both the GFP mean fluorescence level and the percentage of GFP-positive cells significantly increased at 72 hpi post-DENV infection compared with the mock-infected group (Figures 1C,D). To further confirm the EBV viral replicating status during DENV superinfection, mRNA levels of viral immediate-early genes BZLF1 and BRLF1 as well as the reporter GFP were quantified (Figure 1E). In consistency with the increment of EBV genome copy number and GFP protein level, the transcription level of both BZLF1 and BRLF1 started to increase at 36 hpi in DENV-2-infected EBV + Akata cells.

### Wortmannin Treatment Blocks DENV-2 Infection-Induced EBV Reactivation

**FIGURE 2:**
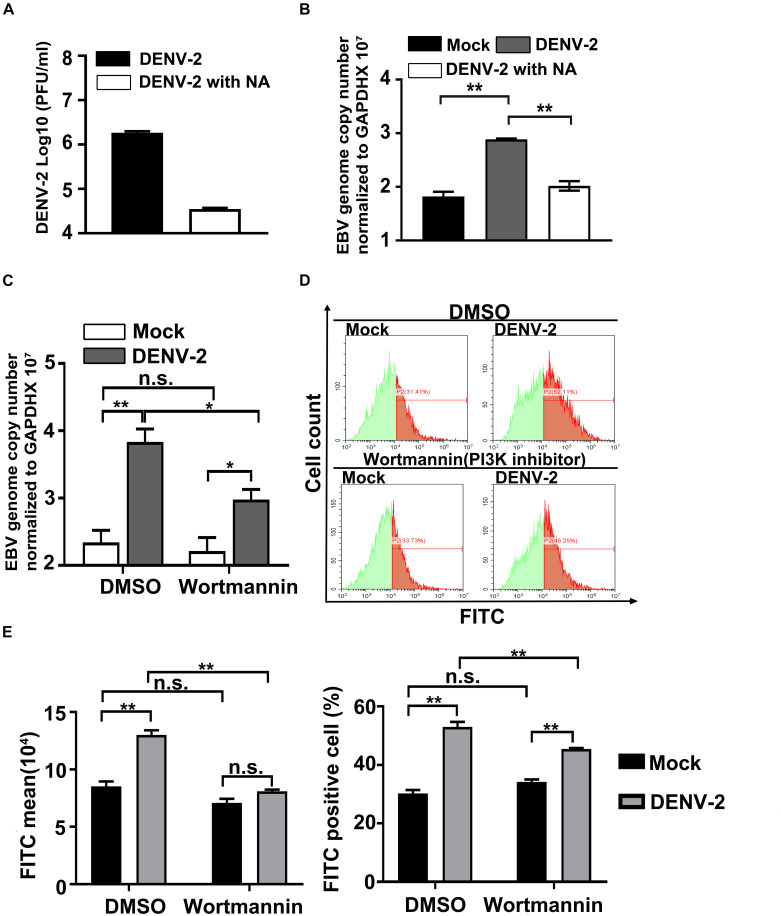
Wortmannin treatment blocks DENV-2 infection-induced Epstein–Barr virus (EBV) reactivation. **(A)** A neutralizing antibody (NA) was mixed with the DENV-2 virus to neutralize infectious viral particles (DENV-2 with NA). The neutralization effect was confirmed by plaque assay. **(B)** EBV + Akata cells were mock-infected or infected with non-neutralized (DENV-2) or neutralized DENV-2 (DENV-2 with NA). At 72 hpi, relative EBV genome load was quantified. **(C,D)** Mock- or DENV-2-infected EBV + Akata cells were treated with DMSO or 0.2 μM wortmannin. At 72 hpi, relative EBV genome load was determined **(C)**. Green fluorescent protein (GFP) intensity of the four experimental groups was quantified by flow cytometry. The representative result of three independent experiments was shown in panel **D**, and quantification and statistical analysis of mean GFP value and percentage of GFP-positive cells were shown in panel **E**. *p* < 0.01 was marked as ** and *p* < 0.05 was marked as *.

To confirm the central role of DENV-2 infection in the above observations, a neutralizing mAb against DENV-2 was employed to block its infectivity. Incubation with the antibody reduced the infectious titer of DENV-2 in the inoculum by more than two orders of magnitude, indicating the effectiveness of this approach (Figure 2A). When the partially neutralized virus was used to infect EBV + Akata cells, a significant reduction in its ability to induce EBV genome copy number increment was detected compared with the non-neutralized DENV-2 virus group (Figure 2B). The above results supported a direct role of DENV-2 infection in EBV reactivation in EBV + Akata cells.

Previous research has reported that DENV infection can activate AKT and phosphoinositide 3-kinase (PI3K) pathway to block cell apoptosis (Lee et al., 2005). Interestingly, the activation of PI3K pathways is known to reactivate EBV in B cells (Darr et al., 2001; Feng et al., 2004; Iwakiri and Takada, 2004; Morrison et al., 2004; Oussaief et al., 2009; Goswami et al., 2012). The assumption that DENV-2 infection in EBV + Akata cells leads to EBV reactivation through its manipulation of PI3K signaling naturally arose. To test this hypothesis, DENV-2-infected or uninfected EBV + Akata cells were treated with DMSO or wortmannin, a well-characterized PI3K inhibitor. At 72 hpi, while DENV-2 infection induced EBV reactivation in the DMSO-treated EBV + Akata cells, treatment with wortmannin significantly reduced cell-associated EBV genome copy number (Figure 2C). Moreover, both total GFP expression level and the percentage of GFP-positive cells in DENV-2-infected EBV + Akata cells were found to be reduced by wortmannin treatment (Figures 2D,E). By contrast, wortmannin treatment in mock-infected group had no significant effect on both EBV genome copy number and GFP expression (Figures 2C–E). Note that blockage of EBV reactivation during dengue infection by wortmannin was not complete (Figure 2C), implying that multiple signaling pathways may signal for EBV reactivation during dengue infection. These results suggested that wortmannin efficiently blocked DENV-2-induced EBV reactivation in EBV + Akata cells.

### Elevated Epstein–Barr Virus Level in Human Peripheral Blood Mononuclear Cells During Acute Dengue Virus Infection

**FIGURE 3:**
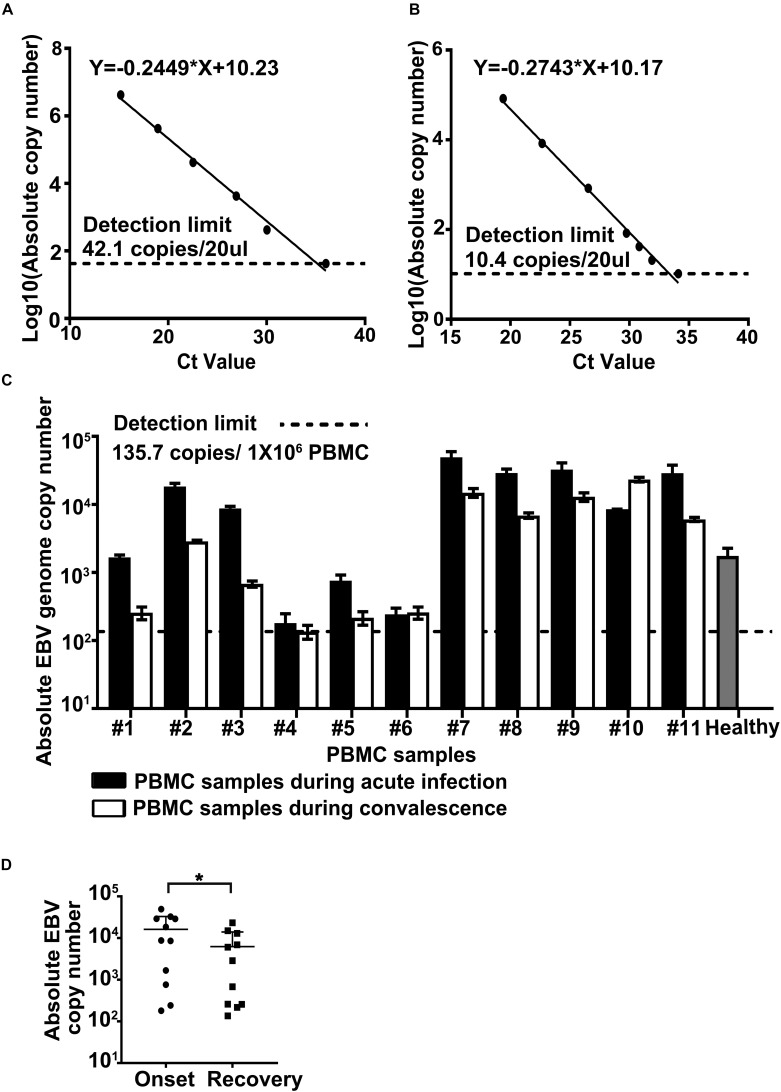
Determination of Epstein–Barr virus (EBV) genome copy number in the peripheral blood mononuclear cells (PBMCs) from dengue patients. Plasmid containing *Bam*HI W region of EBV genome **(A)** or total DNA from Namalwa cells **(B)** served as reference standards in qPCR. Detection limits were given in exact numbers and marked by black dashed lines. Calculation equations were given on top of each chart. **(C)** Absolute EBV genome copy numbers in the PBMCs collected during the acute phase of dengue infection (the black column) or at the time of disease resolution or recovery (the white column) were determined by qPCR, as elaborated in section “Materials and Methods.” Cases 1–6 were in the non-elderly group, and cases 7–11 were in the elderly group. Healthy control (the gray column) was an average of EBV genome level in PBMCs collected from five individuals aged between 23 and 27 years. The statistical analysis of the EBV genome load in samples collected at the time of disease onset vs. that in samples collected at the time of recovery was shown in panel **D**, *p* = 0.0416.

The above results of *in vitro* experiments led us to investigate the changes of EBV level in dengue patients. In total, 11 pairs of PBMC samples from dengue patients were collected (Supplementary Table 2). The first sample was collected during hospitalization and the second one was collected when the patients were in convalescence or recovered. Here, six of 11 cases were individuals younger than 60-year old (non-elderly group, cases 1–6), and five of 11 cases were individuals older than 60-year old (elderly group, cases 7–11). These patients received hemocoagulase, etamsylate, and/or phosphatidylcholine to prevent bleeding and liver injury and other supportive treatments such as rehydration. All patients recovered from dengue diseases after hospitalization. By determination of EBV copy number in the isolated PBMC DNA by qPCR method, the following results were revealed (Figure 3).

Most samples tested positive for EBV DNA, except that the EBV genomic copy numbers in both samples of case 4 (#4) were close to or below the detection limit, consistent with the high prevalence of this virus. We found that the overall PBMC-associated EBV copy number in the elderly group was significantly higher than that in the non-elderly group, in accordance with previous reports of the impaired host immunity against human herpesviruses (HHVs) in old age (Khan et al., 2004; Thomasini et al., 2017).

For the non-elderly group, four out of six had significantly higher PBMC-associated EBV genomic copy number at the time of disease onset; for the elderly group, four out of five experienced an increment of EBV DNA level in their PBMCs during their dengue infection episodes (Figure 3C). In summary, among all 11 pairs of patient samples involved in this study, significantly raised PBMC-associated EBV genomic copy number at the time of dengue fever/hospitalization was evident in eight pairs of samples. Further analysis of the EBV viral genome load in samples collected during disease onset vs. that in samples collected during the recovery period confirmed a statistical difference between these two groups of samples with regard to EBV burden (Figure 3D; Student’s *t*-test, paired, two-tailed, *p*-value < 0.05). The above results agreed with our findings in *in vitro* systems (Figures 1, 2), suggesting a potential *in vivo* interplay between these two viruses.

All PBMC samples tested negative for the presence of other HHVs preferentially infecting lymphocytes, including HHV-6, HHV-7, and human cytomegalovirus (HCMV), under our laboratory settings (data not shown). More sensitive techniques may be needed to investigate those viruses.

### Pre-existing Epstein–Barr Virus Facilitates DENV-2 Infection in Both Direct and Indirect Ways

**FIGURE 4:**
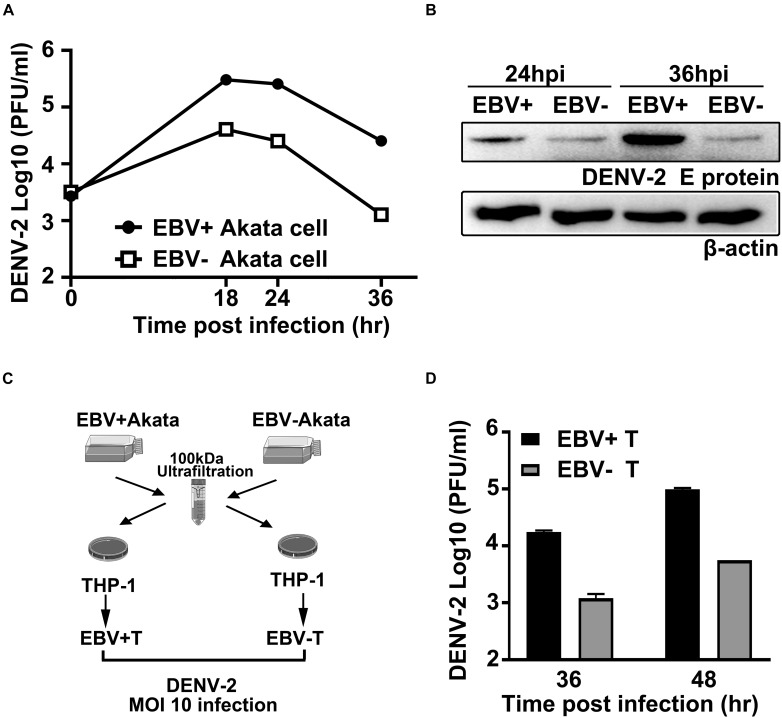
Preexisting Epstein–Barr virus (EBV) infection facilitates DENV-2 propagation in Akata and THP-1 cells. **(A,B)** Equivalent amount of EBV + Akata or EBV- Akata cells were mock-infected or infected with DENV-2 at a multiplicity of infection (MOI) of 10. Growth kinetics of DENV-2 were determined by plaque assay and plotted as in panel **A**. DENV-2 E protein expression level at 24 and 36 hpi was quantified by immune blot **(B)**. **(C)** Schematics illustration of the experimental workflow. Low-molecular weight fractions (<100 kDa) of the culture supernatants from EBV + and EBV- Akata cells were incubated with THP-1 cells for 8 h. Differently primed THP-1 cells were referred to as EBV + T and EBV- T, respectively, in panel **D**. **(D)** The primed THP-1 cells were then infected with DENV-2 at a MOI of 10, and DENV-2 titers in the culture medium at 36 and 48 hpi were determined by plaque assay.

We then proceeded to investigate the effect of persistent EBV infection on DENV propagation. To this aim, the EBV + and EBV- Akata cells were parallelly infected with DENV-2. About 10-fold more DENV-2 infectious particles were produced from EBV + Akata cells than that from EBV- Akata cells at all time points tested (Figure 4A). The intracellular DENV-2 E protein level was also higher in EBV + Akata cells than that in EBV- Akata cells (Figure 4B). To expand the spectrum of this observation, the propagation of ZIKV, another flavivirus, and recombinant vesicular stomatitis virus (VSV-GFP), a rhabdovirus, in these cells was evaluated. ZIKV showed similar advantageous growth in EBV + Akata cells (Supplementary Figure 1A) and in the THP-1 cells stimulated with EBV + Akata cell supernatants (Supplementary Figure 1B). In contrast, VSV-GFP had no apparent growth preference between the EBV + and EBV- Akata cells (Supplementary Figure 1C), which raised an intriguing question whether the preexisting EBV infection in the intracellular environments in Akata cells benefits only specific virus species or families.

Since monocytes are believed to be one of the primarily targeted cells of DENV-2, we investigated whether the preexisting EBV infection in B lymphocytes could affect DENV-2 replication in monocytes indirectly. Culture media from EBV + /EBV- Akata cells were separated into two fractions using 100 kDa nominal molecular weight limit (NMWL) ultrafiltration tubes. The two fractions were used to simulate the THP-1 cells before DENV-2 infection (Figure 4C). The retained fraction from EBV + or EBV- Akata cell culture medium did not show a different effect on DENV-2 propagation in THP-1 cells (Supplementary Figure 2). On the other hand, the DENV-2 titers in THP-1 cells pretreated with the <100 kDa passing-through fraction collected from EBV + Akata cells (EBV + T) were significantly higher than those pretreated with the <100 kDa fraction collected from EBV- Akata cells (EBV-T) at 36 and 48 hpi (Figure 4D). Together, these results suggested that the ongoing EBV infection could prime both EBV-containing B cells and monocytes for DENV-2 propagation *in vitro*, and coinfection in the same host cells was not a prerequisite for such priming effects.

### Inhibition of EBV Replication Abolishes Its Promoting Effect on DENV-2 Replication

**FIGURE 5:**
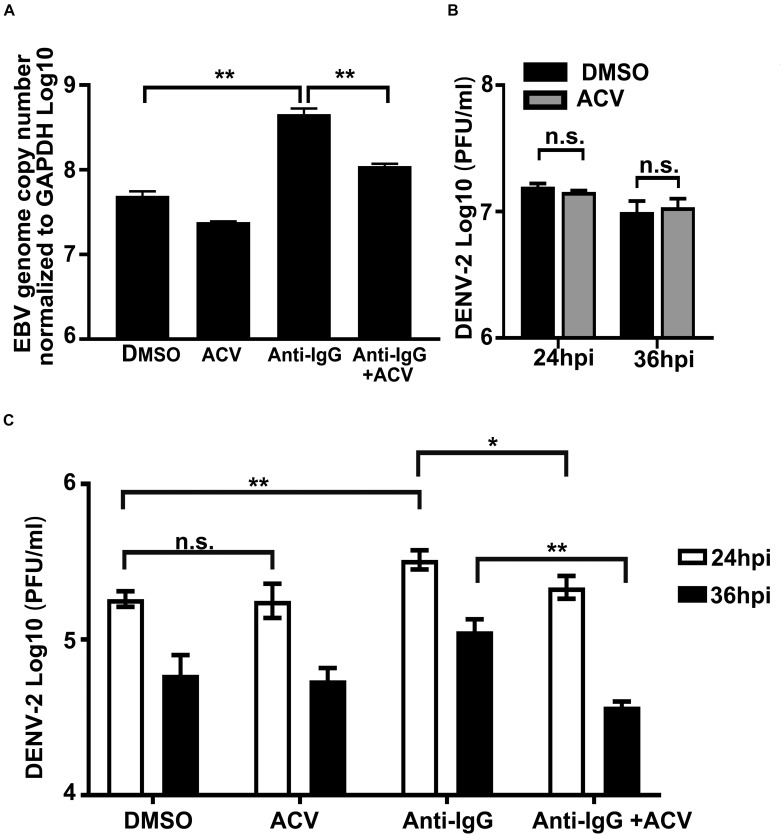
Inhibition of Epstein–Barr virus (EBV) replication in Akata cells abolishes its enhancement effect on DENV-2 replication. **(A)** EBV + Akata cells were treated with dimethyl sulfoxide (DMSO), acyclovir (ACV), anti-human IgG antibody and DMSO, or anti-human IgG and ACV as described in section “Materials and Methods.” Relative EBV genome copy number was measured by qPCR. **(B)** EBV- Akata cells were infected with DENV-2 at a multiplicity of infection (MOI) of 10, DENV-2 titer in culture medium at 24 and 36 hpi was determined by plaque assay. **(C)** The four groups from panel **A** were infected with 10 MOI of DENV-2. At 24 and 36 hpi, DENV-2 yields from the infected EBV + Akata cells were titrated by plaque assay. *p* < 0.01 was marked as ** and *p* < 0.05 was marked as *.

To establish a direct linkage between EBV replication/reactivation and its promotive effect on DENV-2 infection, a series of experiments were performed (Figure 5). Firstly, EBV + Akata cells were treated with anti-human IgG antibody to induce EBV reactivation, and non-induced EBV + Akata cells served as the control group, then ACV or DMSO was applied to the reactivated and the control EBV + Akata cells, respectively. Cellular DNA of the above four groups was isolated, and EBV genome copy number was determined (Figure 5A). The anti-human IgG treatment significantly increased EBV genome copy number in EBV + Akata cells, indicating the successful reactivation of the virus (Takada, 1984; Takada and Ono, 1989; Zhang et al., 2017). Moreover, treatment with ACV efficiently restricted the basal level of EBV replication as well as the EBV reactivation/replication upon anti-human IgG stimulation. To exclude the effect of ACV on DENV-2 infection, DENV-2 was used to infect EBV- Akata cells in the presence or absence of ACV, and we found no inhibitory effect by ACV on DENV-2 virus yield (Figure 5B).

Next, the four experimental groups under the same treatment conditions as in Figure 5A were infected with DENV-2 at a MOI of 10, and culture medium was collected for DENV-2 virus titration. As shown in Figure 5C, DENV-2 virus production in the culture medium peaked at 24 hpi in all groups and slightly declined at 36 hpi. At 24 hpi, the DENV-2 yields from the EBV + Akata/DMSO-treated group and EBV + Akata/ACV-treated group were indistinguishable. In contrast, the EBV + Akata/anti-IgG group was more permissive to DENV-2 replication, and the virus production from this group was significantly higher than that from all other groups at both time points investigated. Note that inhibition of EBV by ACV reduced DENV-2 virus production (Figure 5C; comparison of the EBV + Akata/anti-IgG + ACV group and the EBV + Akata/anti-IgG group, *p* < 0.05). The above results implied that the reactivation/replication of EBV in EBV + Akata cells positively correlated with facilitated DENV-2 replication.

## Discussion

Epstein–Barr virus has one of the highest global infection rates and is persistently infecting more than 90% of the population worldwide (Young et al., 2016). Previously, studies on EBV have intensively focused on its strong correlations with various human cancers (Münz, 2019) and some on its coinfection with a secondary human pathogen such as HCMV, HIV, and various respiratory pathogens, e.g., influenza virus, adenovirus, severe acute respiratory syndrome coronavirus 2 (SARS-CoV-2), and *Mycoplasma pneumoniae* (Breinig et al., 1987; Liebowitz, 1998; Weinberg et al., 2005; Wang et al., 2010; Jakovljevic et al., 2015; García-Martínez et al., 2020). To our knowledge, this is the first piece of study examining the dynamic changes of EBV status during DENV infection both *in vitro* and in clinical samples from dengue patients.

We firstly demonstrated that DENV-2 infection of EBV + Akata cells led to EBV reactivation (Figure 1); the PI3K signaling pathway played a role in DENV-2’s reactivation of EBV (Figures 2C,D). Whether other pathways were involved in the above scenario needed additional investigations. Interestingly, a previous report claimed that the malaria infection induces the EBV replication in a manner of initiating the B-cell receptor (BCR) signal pathway through the cysteine-rich interdomain region 1α (CIDR1α) of malaria (Chêne et al., 2007).

By examining the EBV DNA copy number in PBMCs from dengue patients and healthy donors (Figure 3), an elevated cell-associated EBV DNA load in PBMCs was found to be strongly associated with the dengue infection symptomatic phase in eight out of 11 paired human PBMC samples. This intriguing observation agreed with the results of our *in vitro* assays, supporting the possibility that EBV was reactivated *in vivo* by DENV infection. However, in spite of the efforts made to detect EBV gene expression (EBNA1, LMP1, and BZLF1) in the PBMC samples of dengue patients, no stable detection was found (data not shown). The possible explanations could be that most EBV-harboring cells in PBMCs are in latency 0, or that the reactivation event of EBV *in vivo* during dengue infection was very transient, partially due to host immunity. However, other explanations, e.g., EBV-harboring B cells were preferentially amplified during DENV infection, are possible and should be investigated by more elaborate methodologies.

We also found that EBV infection facilitated DENV-2 propagation, and this effect did not require the same cell to be coinfected by the two viruses. The molecular mechanisms behind are not fully understood herein, but an association with the secreted biomolecules from EBV + Akata cells was implied. Finally, through molecular approaches, a positive correlation between EBV replication and DENV-2 replication was established in EBV + Akata cells (Figure 5C). In all, the above *in vitro* and *in vivo* observations signified an unneglectable role of EBV during acute DENV infection, highlighting the importance of further in-depth investigations.

## Data Availability Statement

The raw data supporting the conclusions of this article will be made available by the authors, without undue reservation.

## Ethics Statement

The studies involving human participants were reviewed and approved by Institutional Review Broad of School of Medicine, Sun Yat-sen University; Ethics Review Board of Guangzhou Eighth People’s Hospital. The patients/participants provided their written informed consent to participate in this study. Written informed consent was obtained from the individual(s) for the publication of any potentially identifiable images or data included in this article.

## Author Contributions

PX conceived the project. X-MD, L-ZZ, X-YL, and DL performed the experiments. PX, Z-YL, and X-MD provided critical intellectual input to the study, analyzed most data, and prepared and wrote the manuscript. L-ZZ, F-CZ, and LY provided the crucial dengue patient samples and the neutralizing mAb against DENV-2. HZ provided the cell lines. All authors read and approved the final manuscript.

## Conflict of Interest

The authors declare that the research was conducted in the absence of any commercial or financial relationships that could be construed as a potential conflict of interest.
