# Supplementary material for: In vitro Studies and Clinical Observations Imply a Synergistic Effect Between Epstein-Barr Virus and Dengue Virus Infection
Source: Front Microbiol. 2021 Jun 18;12:691008. doi: 10.3389/fmicb.2021.691008 (PMC8249608; doi:10.3389/fmicb.2021.691008)
Supplement: Supplementary file 1 [file Data_Sheet_1.PDF]

Supplementary Table 1 Primers and Probes for EBV qRT-PCR

Amplification target	Primers and Probe	Sequences and labels (5′-3′)
BamHI W	F	CCCAACACTCCACCACACC
	R	TCTTAGGAGCTGTCCGAGGG
	Probe	FAM-CACACACTACACACACCCACCCGTCTC-TARMA
EBNA1	F	TAGATTTGCCTCCCTGGTTTC
	R	CATCTCCATCACCTCCTTCATC
GAPDH	F	GAAGGTGAAGGTCGGAGTC
	R	GAAGATGGTGATGGGATTTC
GFP	F	AAGCTGACCCTGAAGTTCATCTGC
	R	CTTGTAGTTGCCGTCGTCCTTGAA
BZLF	F	CACCTCAACCTGGAGACAATTC
	R	GCTAGCTGTTGTCCTTGGTTAG
BRLF	F	GGAGATGGCTGACACTGT
	R	GACTCTAGTGTTGTGGTCAGTTC
18S rRNA	F	TGTGCCGCTAGAGGTGAAATT
	R	TGGCAAATGCTTTCGCTTT

Supplementary Table 2 The information of human PBMC samples

Case number	Age	Sample type	Date of Collection	Interval Days of Sampling	DENV-2 viremia (copy number/ml serum)
Case#1	54	Symptomatic period	2017/8/26	48	33*
		Recovered/discharged	2017/10/14		ND
Case#2	34	Symptomatic period	2017/8/8	46	25*
		Recovered/discharged	2017/9/24		ND
Case#3	25	Symptomatic period	2017/10/13	44	26*
		Recovered/discharged	2017/11/27		ND
Case#4	22	Symptomatic period	2017/9/29	6	35*
		Recovered/discharged	2017/10/5		ND
Case#5	19	Symptomatic period	2017/10/13	45	40*
		Recovered/discharged	2017/12/3		ND
Case#6	54	Symptomatic period	2017/8/8	50	40*
		Recovered/discharged	2017/10/13		ND
Case#7	77	Symptomatic period	2014/9/26	4	1.76×10^{3}
		Recovered/discharged	2014/9/30		3.18×10^{2}
Case#8	98	Symptomatic period	2014/9/26	12	ND
		Recovered/discharged	2014/10/8		ND
Case#9	73	Symptomatic period	2014/9/26	4	1.92×10^{5}
		Recovered/discharged	2014/9/30		ND
Case#10	66	Symptomatic period	2014/9/26	4	1.92×10^{8}
		Recovered/discharged	2014/9/30		negative
Case#11	73	Symptomatic period	2014/9/26	18	ND
		Recovered/discharged	2014/10/14		ND

Ct values from the clinical qRT-PCR diagnosis test.
ND: not determined.
