# Supplementary figures and images for: In vitro Studies and Clinical Observations Imply a Synergistic Effect Between Epstein-Barr Virus and Dengue Virus Infection
Source: Front Microbiol. 2021 Jun 18;12:691008. doi: 10.3389/fmicb.2021.691008 (PMC8249608; doi:10.3389/fmicb.2021.691008)

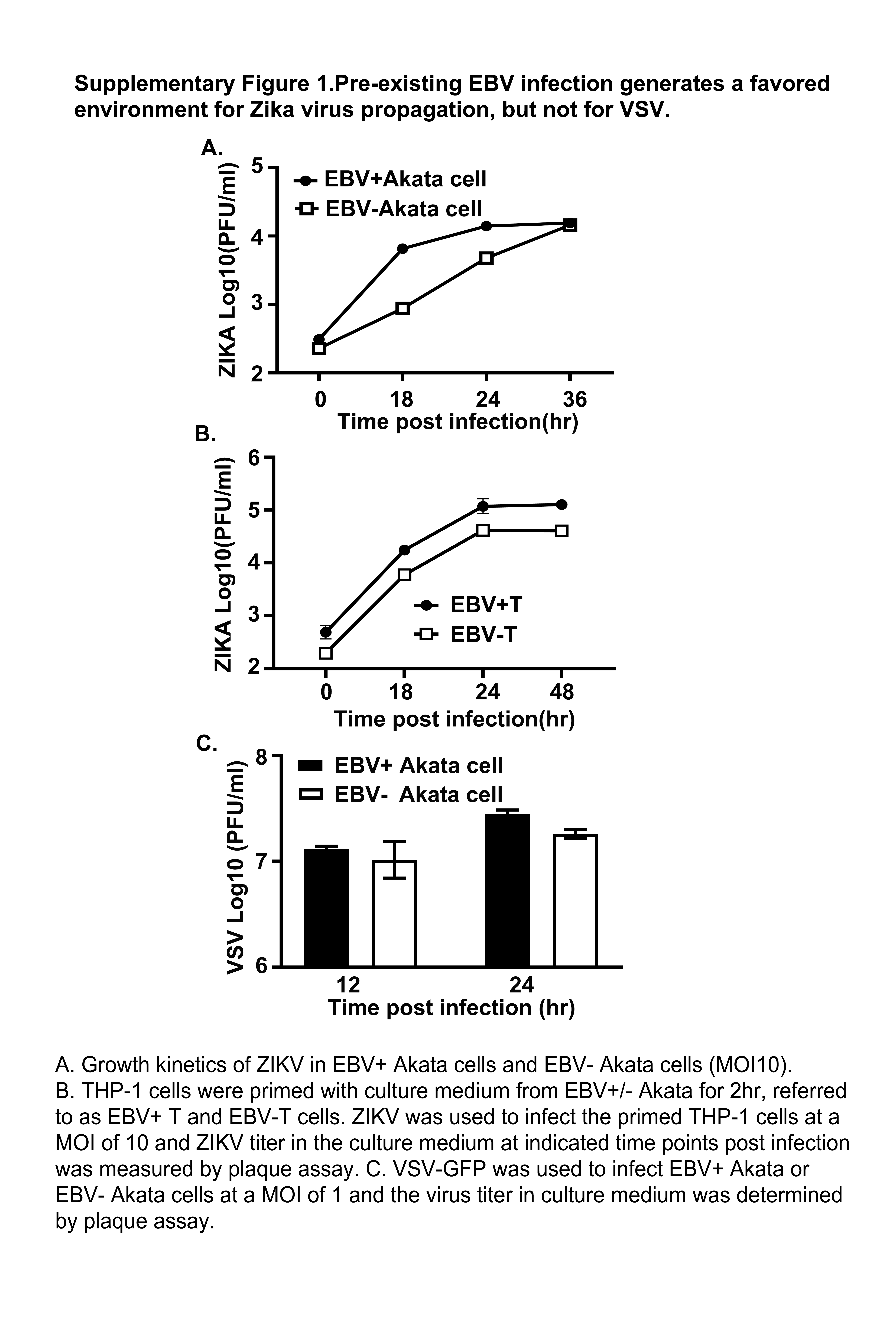

Supplement: Supplementary file 2 [file Image_1.JPEG]

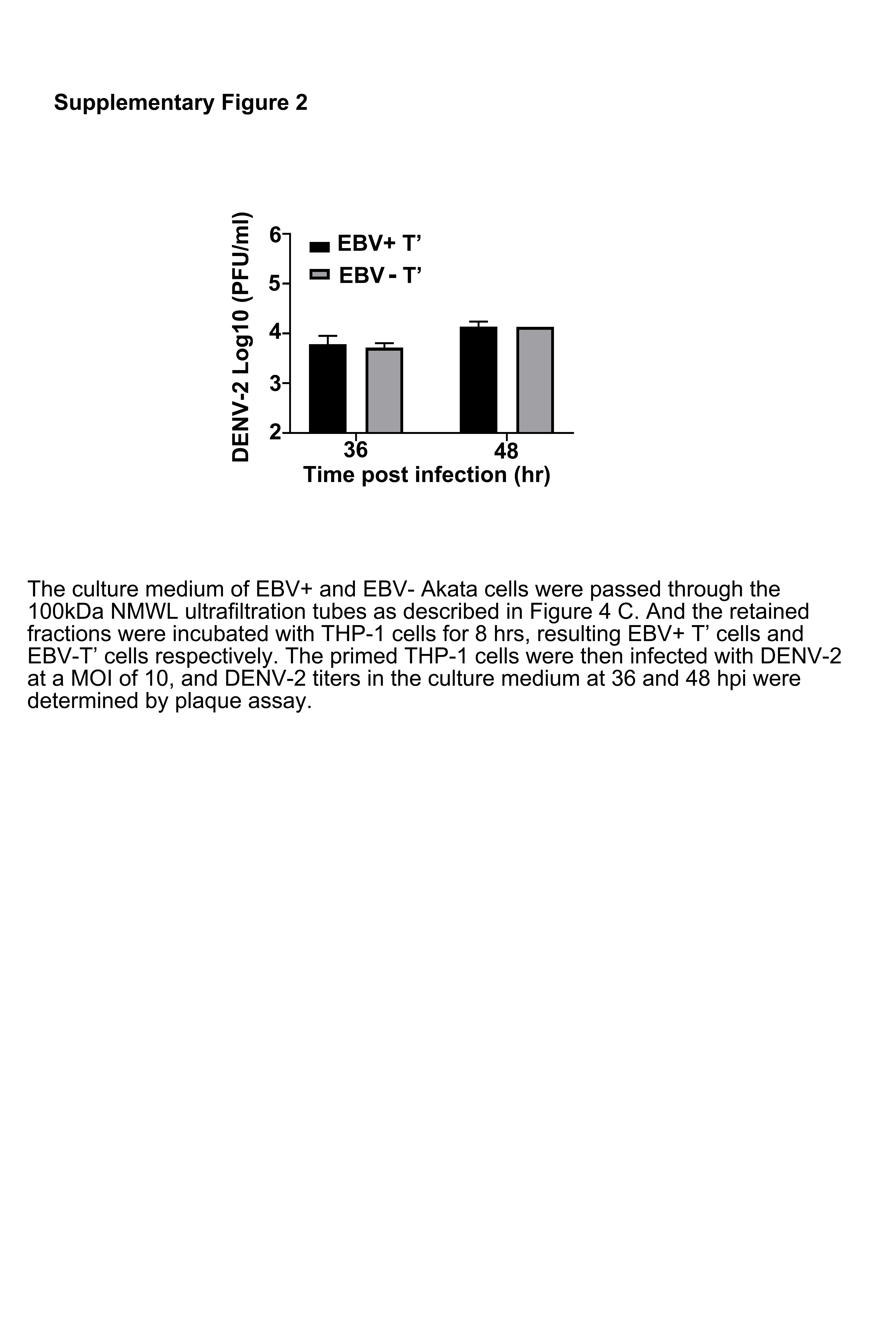

Supplement: Supplementary file 3 [file Image_2.JPEG]
